# Cell Type-Specific Genetic Manipulation and Impaired Circadian Rhythms in *Vip* ^*tTA*^ Knock-In Mice

**DOI:** 10.3389/fphys.2022.895633

**Published:** 2022-05-03

**Authors:** Yubo Peng, Yusuke Tsuno, Ayako Matsui, Yuichi Hiraoka, Kohichi Tanaka, Shin-ichi Horike, Takiko Daikoku, Michihiro Mieda

**Affiliations:** ^1^ Department of Integrative Neurophysiology, Graduate School of Medical Sciences, Kanazawa University, Kanazawa, Japan; ^2^ Laboratory of Molecular Neuroscience, Medical Research Institute, Tokyo Medical and Dental University (TMDU), Tokyo, Japan; ^3^ Division of Integrated Omics Research, Research Center for Experimental Modeling of Human Disease, Kanazawa University, Kanazawa, Japan; ^4^ Division of Animal Disease Model, Research Center for Experimental Modeling of Human Disease, Kanazawa University, Kanazawa, Japan

**Keywords:** circadian rhythm, biological clock, vasoactive intestinal peptide, suprachiasmatic nucleus, Tet system, genetically engineered mice, neural circuit, cerebral cortex

## Abstract

The suprachiasmatic nucleus (SCN), the central circadian clock in mammals, is a neural network consisting of various types of GABAergic neurons, which can be differentiated by the co-expression of specific peptides such as vasoactive intestinal peptide (VIP) and arginine vasopressin (AVP). VIP has been considered as a critical factor for the circadian rhythmicity and synchronization of individual SCN neurons. However, the precise mechanisms of how VIP neurons regulate SCN circuits remain incompletely understood. Here, we generated *Vip* ^*tTA*^ knock-in mice that express tetracycline transactivator (tTA) specifically in VIP neurons by inserting tTA sequence at the start codon of *Vip* gene. The specific and efficient expression of tTA in VIP neurons was verified using EGFP reporter mice. In addition, combined with *Avp-Cre* mice, *Vip* ^*tTA*^ mice enabled us to simultaneously apply different genetic manipulations to VIP and AVP neurons in the SCN. Immunostaining showed that VIP is expressed at a slightly reduced level in heterozygous *Vip* ^*tTA*^ mice but is completely absent in homozygous mice. Consistently, homozygous *Vip* ^*tTA*^ mice showed impaired circadian behavioral rhythms similar to those of *Vip* knockout mice, such as attenuated rhythmicity and shortened circadian period. In contrast, heterozygous mice demonstrated normal circadian behavioral rhythms comparable to wild-type mice. These data suggest that *Vip* ^*tTA*^ mice are a valuable genetic tool to express exogenous genes specifically in VIP neurons in both normal and VIP-deficient mice, facilitating the study of VIP neuronal roles in the SCN neural network.

## Introduction

The SCN is a heterogeneous structure made up of various types of neurons (Antle and Silver, 2005). Almost all SCN neurons contain γ-aminobutyric acid (GABA) as a neurotransmitter. These neurons can be differentiated by the co-expression of specific peptides (Welsh et al., 2010). These include AVP-producing neurons located predominantly in the dorsomedial part or the shell of the SCN, as well as vasoactive intestinal peptide (VIP)-producing neurons and gastrin-releasing peptide (GRP)-producing neurons in the ventrolateral part or the core of the SCN. VIP is expressed in approximately 10% of SCN neurons (Abrahamson and Moore, 2001; Herzog et al., 2017). It is the most important contributor to the synchronization among SCN neurons (Harmar et al., 2002; Colwell et al., 2003; Aton et al., 2005; Maywood et al., 2006). VIP neurons receive direct projections from the retina, and VIP has been implicated in the photoentrainment of the central circadian clock of the SCN (Abrahamson and Moore, 2001; Colwell et al., 2003; Jones et al., 2015, 2018; Vosko et al., 2015). Therefore, mice deficient in VIP (*Vip* ^*−/−*^) or its receptor (*Vipr2* ^*−/−*^) demonstrate a variety of abnormalities in the circadian rhythmicity and synchrony, including arrhythmicity, multiple circadian periods, shortened free-running period, and reduced responsiveness to the light (Harmar et al., 2002; Colwell et al., 2003; Aton et al., 2005). However, the precise mechanisms of how VIP neurons regulate SCN circuits remain incompletely understood. Also, other neuropeptides, such as AVP and GRP, may play some roles in the intercellular communication of SCN neurons (Li et al., 2009; Maywood et al., 2011; Ono et al., 2016).

Genetic manipulation specific to VIP neurons is a powerful approach to studying these neurons’ functions. So far, genetically modified mice utilizing the Cre/loxP system and FLP/FRT system have been generated, namely, *Vip-ires-Cre* and *Vip-ires-Flp* mice, respectively (Taniguchi et al., 2011; He et al., 2016). The Tet system is another genetic engineering system in which tetracycline transactivator (tTA) binds tetracycline-responsive element (TRE) and activates the gene downstream of TRE (Gossen and Bujard, 1992; Aiba and Nakao, 2007). Moreover, the tTA activity can be turned off (Tet-off system) or turned on (reverse tTA, Tet-on system) in the presence of tetracycline or its analogue doxycycline. Here, we established a *Vip* ^*tTA*^ knock-in mouse line in which tTA2 (Urlinger et al., 2000) is expressed specifically in VIP neurons. In combination with a series of genetically modified mice and viral vectors equipped with TRE-driven gene expression, *Vip* ^*tTA*^ mice can express various exogenous genes specifically in VIP neurons. Furthermore, because the transcription of the endogenous *Vip* coding sequence was blocked in the *Vip* ^*tTA*^ allele, homozygous mice of the line behaved as *Vip*-deficient mice. Thus, this new mouse line provides a valuable tool for the functional study of VIP neurons.

## Materials and Methods

### Animals

**FIGURE 1:**
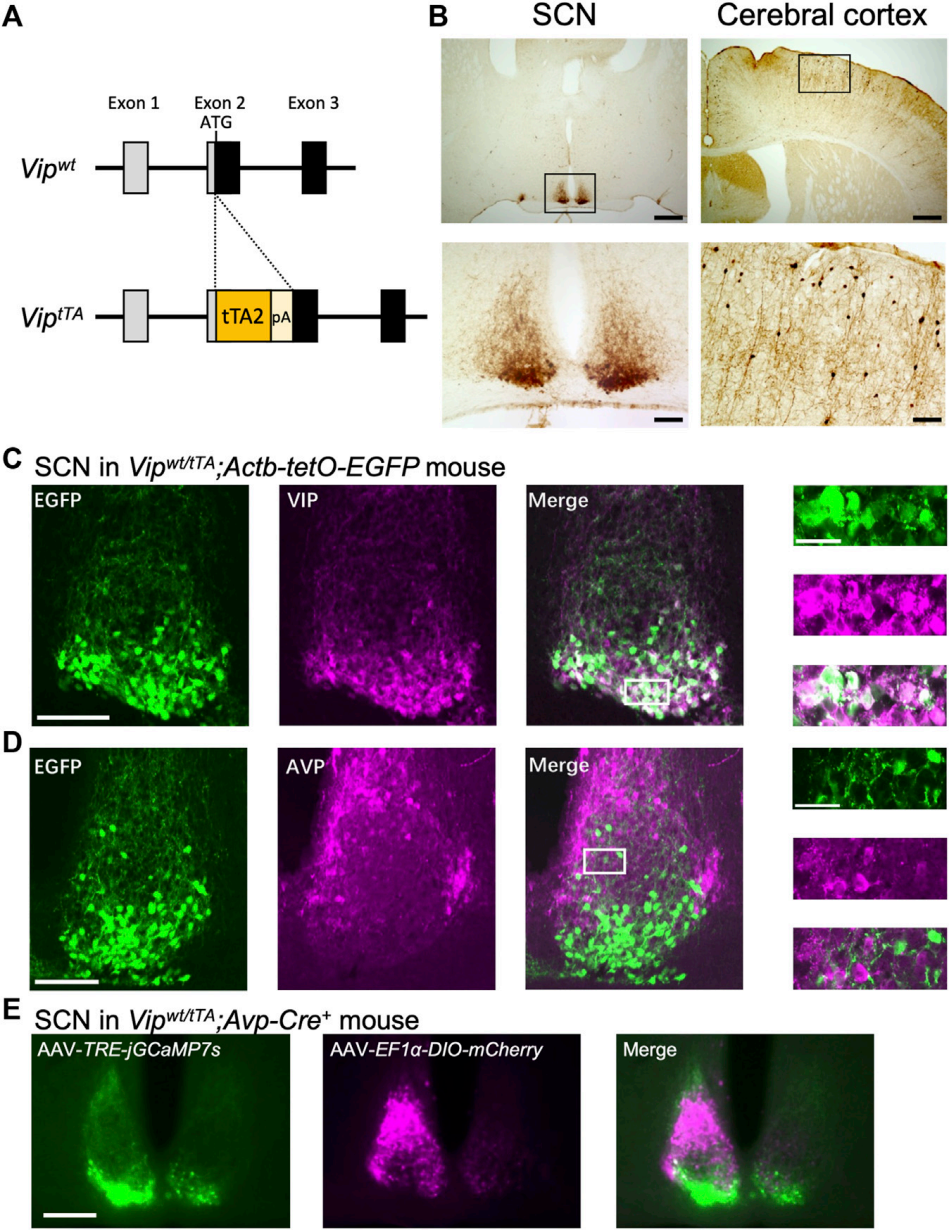
Generation and validation of *Vip* ^*tTA*^ knock-in mice. **(A)** Targeting strategy for generating *Vip* ^*tTA*^ mice. A *tTA2-polyA* cassette was inserted at the start codon of *Vip* gene in exon 2. **(B)** tTA-mediated EGFP expression was mostly restricted in the SCN and cerebral cortex in *Vip* ^*wt/tTA*^ mice crossed with *Actb-tetO-EGFP* reporter mice. Coronal brain sections were immunostained for GFP in brown. Lower panels (scale bar, 100 μm) are magnified images of regions indicated by rectangles in upper panels (scale bar, 500 μm). **(C–D)** Coronal brain sections prepared from *Vip* ^*wt/tTA*^ *;Actb-tetO-EGFP* mice were immunostained for VIP **(C)** or AVP **(D)** in magenta (middle). Native EGFP fluorescent images are shown on left. The white rectangles in the merged images (scale bar, 100 μm) indicate the regions of the enlarged images (far right; scale bar, 30 μm). For fluorescent immunostaining, mice were pretreated with intracerebroventricular injections of colchicine (40 μg in 1 μl saline) for 48 h before transcardial perfusion of 4% paraformaldehyde fixative. **(E)** Differential labeling of VIP and AVP neurons in the SCN of *Vip* ^*wt/tTA*^ *;Avp-Cre* ^*+*^ mice. AAV*-EF1α-DIO-mCherry* and AAV*-TRE-jGCaMP7s* were injected unilaterally into the SCN in *Vip* ^*wt/tTA*^ *;Avp-Cre* ^*+*^ mice. A representative coronal section of the SCN is shown (scale bar, 200 μm) (n = 3). Note that jGCaMP7s-labeled neurons (left, green) are in the SCN core, while mCherry-labeled neurons (middle, magenta) are in the SCN shell. These two populations of neurons rarely overlap (right).

To generate *Vip* ^*tTA*^ mice, we inserted a *tTA2-polyA* cassette at the start codon of *Vip* gene in its second exon by the CRISPR/Cas9-mediated targeting strategy (Figure 1A). The donor DNA was synthesized, containing *tTA2* cDNA (Urlinger et al., 2000), *SV40 polyA* signal, and 300 bp sequences of the mouse *Vip* gene (NCBI Gene: 22353) 5′ and 3′ to the start codon. C57BL/6J mice were purchased from Japan SLC, Inc. (Shizuoka, Japan). One crRNA targeting exon 2 (5′- TCT​TTT​CAG​AGG​CAC​CGA​GA -3′) of the mouse *Vip* gene was designed using the online sgRNA design tool available at https://crispr.mit.edu/ and purchased from Integrated DNA Technologies (Coralville, IA). The fertilized pronuclear-stage embryos were prepared by *in vivo* fertilization in human tubal fluid medium (ARK Resource; Kumamoto, Japan) with sperms from two C57BL/6J males and oocytes from five superovulated females injected with anti-inhibin serum and human chorionic gonadotropin. Next, the ssODN (40 ng/μl) and the complex of crRNAs (0.61 μM), tracrRNA (0.61 μM), and Cas9 protein (30 ng/μl) (Integrated DNA Technologies) in TE were injected into the nucleus of approximately two hundred pronuclear-stage embryos by microinjection. Embryos were then washed and cultured in potassium simplex optimization medium (ARK Resource) overnight. The obtained ninety-three 2-cell embryos were transferred to recipient mice. Fourteen mice were born and tested for the correct targeting of the *tTA2-polyA* cassette by genomic PCR and sequencing. Six out of 14 mice (F0) had a correct insertion of the cassette, and two F0 mice were bred with C57BL/6J mice to obtain F1 generation. These two lines backcrossed to C57BL/6J mice at least twice were used for further analyses. We did not discriminate between these two lines in this manuscript because they were indistinctive regarding the tTA2 expression and circadian behavior. To evaluate the specific expression of tTA2, *Vip* ^*tTA*^ mice were crossed to *Actb-tetO-EGFP* reporter mice. *Actb-tetO-EGFP* reporter mice were generated from *Actb-tetO-FLEX-EGFP* mice (Aida et al., 2015) by crossing with germ cell-specific *Prdm1-Cre* mice (JAX 008827) to eliminate the Cre-dependency of EGFP expression.

*Avp-Cre* mice were reported previously (Mieda et al., 2015) and used in hemizygous condition. This line is a transgenic mouse harboring a modified BAC transgene, which has an insertion of codon-improved Cre recombinase gene immediately 5′ to the translation initiation codon of exogenous *Avp* gene in the BAC, but without manipulation of the endogenous *Avp* loci in the mouse.

All mice were maintained under a strict 12 h light/12 h dark (LD) cycle in a temperature- and humidity-controlled room and fed ad libitum. All experimental procedures involving animals were approved by the appropriate institutional animal care and use committees of Kanazawa University and Tokyo Medical and Dental University.

### Immunohistochemistry

**FIGURE 2:**
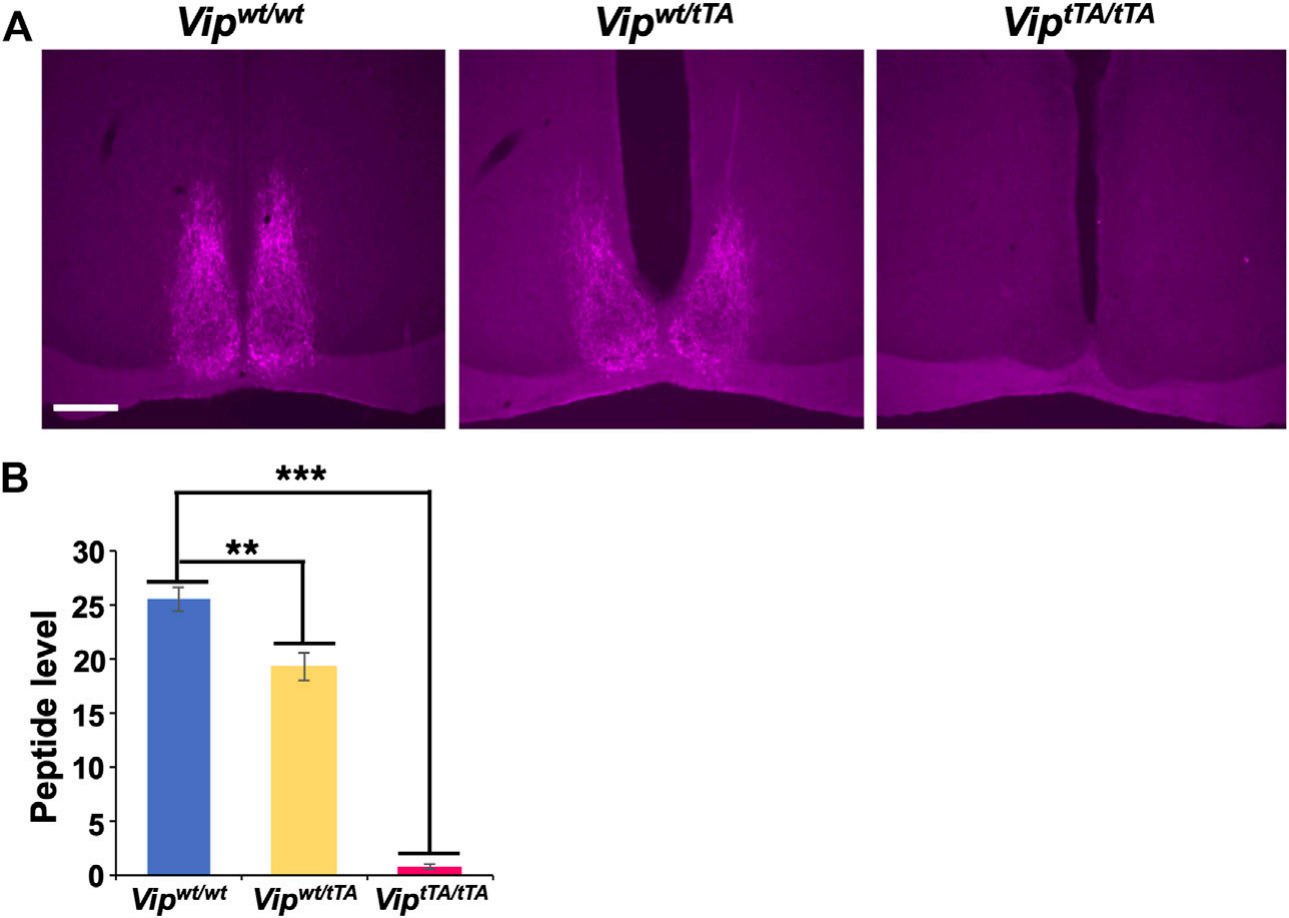
VIP expression is absent in *Vip* ^*tTA/ tTA*^ mice. **(A)** Coronal brain sections containing the SCN prepared from *Vip* ^*wt/wt*^, *Vip* ^*wt/tTA*^, and *Vip* ^*tTA/tTA*^ mice were immunostained for VIP (magenta; scale bar, 200 μm). Representative images are shown. **(B)** VIP immunostaining was slightly reduced in *Vip* ^*wt/tTA*^ (n = 4) compared to *Vip* ^*wt/wt*^ (n = 3) and almost absent in *Vip* ^*tTA/tTA*^ (n = 5) mice. Error bars indicate SEM. **, *p* < 0.005; ***, *p* < 0.001 by one-way ANOVA followed by *post-hoc* Tukey tests.

Immunostaining was performed as described previously (Mieda et al., 2015). Mice were sacrificed at approximately ZT5 by transcardial perfusion of PBS followed by 4% paraformaldehyde fixative. To examine the specificity of tTA2 expression by fluorescent immunostaining (in Figures 1C,D), *Vip* ^*wt/tTA*^ *;Actb-tetO-EGFP* mice were pretreated with intracerebroventricular colchicine injections (40 µg in 1 µl saline) for 48 h before perfusion fixation. Serial coronal brain sections (30 µm thick) were prepared with a cryostat (CM 1860, Leica) and collected in 4 series—one of which was further immunostained. The antibodies used were: rabbit anti-GFP (Thermo Fisher Scientific, 1:1,000), rabbit anti-AVP (Millipore, 1:4,000); rabbit anti-VIP (Immunostar, 1:1,000); biotinylated goat anti-rabbit IgG antibody (Vector Lab, 1:1,000), and Alexa 488-conjugated goat anti-rabbit IgG (Molecular Probes, 1:1,000). The expression of EGFP was detected by its native fluorescence for fluorescent immunostaining (Figures 1C,D). The VIP expression levels were quantified by Photoshop (Adobe) as follows (Figure 2B). First, the images were transformed to grayscale. Then, the mean intensities of pixels within the SCN were calculated. Finally, the values of the region lateral to the SCN were regarded as background and were subtracted from those of the SCN. For immunohistochemistry using diaminobenzidine (DAB) reactions (Figure 1B), color development was performed using the VECTASTAIN Elite ABC-HRP Kit (PK6100, Vector Lab) and DAB Substrate Kit (SK4100, Vector Lab). For Figures 1A,C,D representative optical section was imaged from each stained section by laser-confocal microscopy (Olympus, FluoView FV10i), then fluorescent cells in the images were counted. For Figures 1B, 2A, immunostaining of EGFP or VIP in sections was observed by epifluorescence or bright-field microscopy (KEYENCE, BZ-9000E).

### Generation and Focal Injection of Recombinant AAV Vectors

The AAV-2 ITR-containing plasmid *pAAV-TRE-ChR2-EYFP* (Addgene #110339, a gift from Dr. Hyungbae Kwon) was modified to construct *pAAV-TRE-jGCaMP7s* by replacing a BamHI-HindIII fragment containing *hChR2-EYFP* cDNA with a BamHI-HindIII fragment containing *jGCaMP7s* cDNA amplified by PCR from the plasmid *pGP-AAV-CAG-FLEX-jGCaMP7s-WPRE* (Addgene #104495, a gift from Dr. Douglas Kim & GENIE Project), using the following primers: 5′- atagga​tccgcc​acc​ATG​ggt​tct​cat​ca -3′ and 5′- gcgaag​ctTCAc​ttc​gct​gtc​atc​att​tg -3′. *pAAV-EF1a-DIO-mCherry* was a gift from Dr. Bryan Roth (Addgene #50462).

Recombinant AAV vectors (AAV2-rh10), AAV-*TRE-jGCaMP7s* and AAV-*EF1α-DIO-mCherry*, were produced using a triple-transfection, helper-free method and purified as described previously (Mieda et al., 2015). The titers of recombinant AAV vectors were determined by quantitative PCR: AAV-*TRE-jGCaMP7s*, 6.3 × 10^11^; and AAV-*EF1α-DIO-mCherry*, 5.2 × 10^12^ genome copies/ml. Stereotaxic injection of AAV vectors into the SCN of *Vip* ^*wt/tTA*^ *;Avp-Cre* ^*+*^ mice was performed as described previously (Maejima et al., 2021). Two weeks after surgery, coronal brain slices (100 µm thick) were prepared, and fluorescence of jGCaMP7s and mCherry was observed as described above.

### Behavioral Analyses

Male and female, *Vip* ^*wt/wt*^, *Vip* ^*wt/tTA*^, and *Vip* ^*tTA/tTA*^ littermates, aged 8–17 weeks, were individually housed in a polycarbonate cage placed in a light-tight box. Spontaneous movements in the homecage were monitored by infrared sensors (O’hara) in 1-min bins as described previously (Mieda et al., 2015). Actogram, activity profile, and χ^2^ periodogram analyses were performed *via* ClockLab (Actimetrics). The free-running period and amplitude (Qp values) were calculated for the last 10 days in constant darkness (DD) by periodogram. The activity onset was calculated from the daily activity profile (average pattern of activity) of the last 7 days in LD using the mean activity level as a threshold.

### Statistics

All results are expressed as mean ± SEM. For comparison of three or four groups in Figures 2, 4, one-way ANOVA followed by Tukey post hoc tests were performed. Probability (p) values less than 0.05 were considered to be statistically significant. Only relevant information from the statistical analysis was indicated in the text and figures.

## Results

### Generation of *Vip* ^*tTA*^ Knock-In Mice

We generated knock-in mice that express tTA2 (Urlinger et al., 2000) specifically in VIP neurons. To do this, we employed the CRISPR/Cas9-mediated homologous recombination to target the *Vip* gene of the mouse genome to insert a *tTA2-polyA* cassette at the start codon of *Vip* gene in its exon 2 (*Vip* ^*tTA*^) (Figure 1A). To localize tTA2 activity, we crossed *Vip* ^*wt/tTA*^ mice to *Actb-tetO-EGFP* reporter mice, which express EGFP in the presence of tTA. EGFP expression was mostly restricted in the SCN and the cerebral cortex in these mice (Figure 1B). The cerebral cortex contains a population of GABAergic interneurons that expresses VIP (Taniguchi et al., 2011). In contrast, there were few EGFP + cells in other regions where some neurons express VIP, such as the hippocampus and amygdala. In the SCN, EGFP expression was almost completely colocalized with VIP immunoreactivity (83.76 ± 0.91% of VIP + cells were also EGFP+, 87.11 ± 1.47% of EGFP + cells were also VIP+; counts of three SCN slices each from three mice) (Figure 1C). In contrast, there was almost no overlap with AVP immunoreactivity (2.45 ± 0.59% of AVP + cells were also EGFP+, 5.21 ± 1.16% of EGFP + cells were also AVP+) (Figure 1D). Thus, the expression of tTA2 occurred specifically and efficiently in VIP neurons within the SCN, confirming that *Vip* ^*tTA*^ mice are a useful tool for VIP-neuron-specific genetic manipulations.

### Differential Labeling of Vasoactive Intestinal Peptide and Arginine Vasopressin Neurons in the Suprachiasmatic Nucleus of *Vip* ^*wt/tTA*^ *;Avp-Cre* ^*+*^ Mice

Next, we examined whether *Vip* ^*tTA*^ mice are useful to simultaneously apply different genetic manipulations to VIP and AVP neurons nearby within the SCN. To do so, we crossed *Vip* ^*wt/tTA*^ mice to hemizygous *Avp-Cre* mice (*Avp-Cre* ^*+*^) that express improved Cre recombinase specifically in AVP neurons (Mieda et al., 2015). Then, we focally injected two AAV vectors in the SCN of *Vip* ^*wt/tTA*^ *;Avp-Cre* ^*+*^ mice: AAV-*TRE-jGCaMP7s* and AAV-*EF1α-DIO-mCherry* that express a green fluorescent Ca^2+^ indicator protein jGCaMP7s (Dana et al., 2019) and a red fluorescent protein mCherry in a tTA- and Cre-dependent manner, respectively. As expected, jGCaMP7s was expressed specifically in the SCN shell, where AVP neurons locate, whereas mCherry expression was restricted in the SCN core, where VIP neurons locate (Figure 1E). Furthermore, there was almost no overlap in the expression of these two proteins. Thus, we successfully labeled VIP and AVP neurons differentially within the SCN local circuit.

### Homozygous *Vip* ^*tTA*^ Mice Lack Vasoactive Intestinal Peptide Expression

The *Vip* coding sequence was interrupted by a *tTA2-polyA* sequence in the *Vip* ^*tTA*^ allele, resulting in the expression of tTA2 under the *Vip* promoter. In other words, the *Vip* ^*tTA*^ allele should act equivalently to a *Vip* knockout allele. Therefore, we next examined the VIP expression in heterozygous *Vip* ^*wt/tTA*^ and homozygous *Vip* ^*tTA/tTA*^ mice by immunostaining. We found that VIP-immunoreactivity in *Vip* ^*wt/tTA*^ mice was slightly reduced (∼20%) compared to wild-type mice (*Vip* ^*wt/wt*^) but was completely absent in *Vip* ^*tTA/tTA*^ (Figure 2). These results suggested that the insertion of a *tTA2-polyA* sequence at the translation initiation site completely blocks the expression of VIP peptide.

### Homozygous *Vip* ^*tTA*^ Mice Show Impaired Circadian Rhythms

**FIGURE 3:**
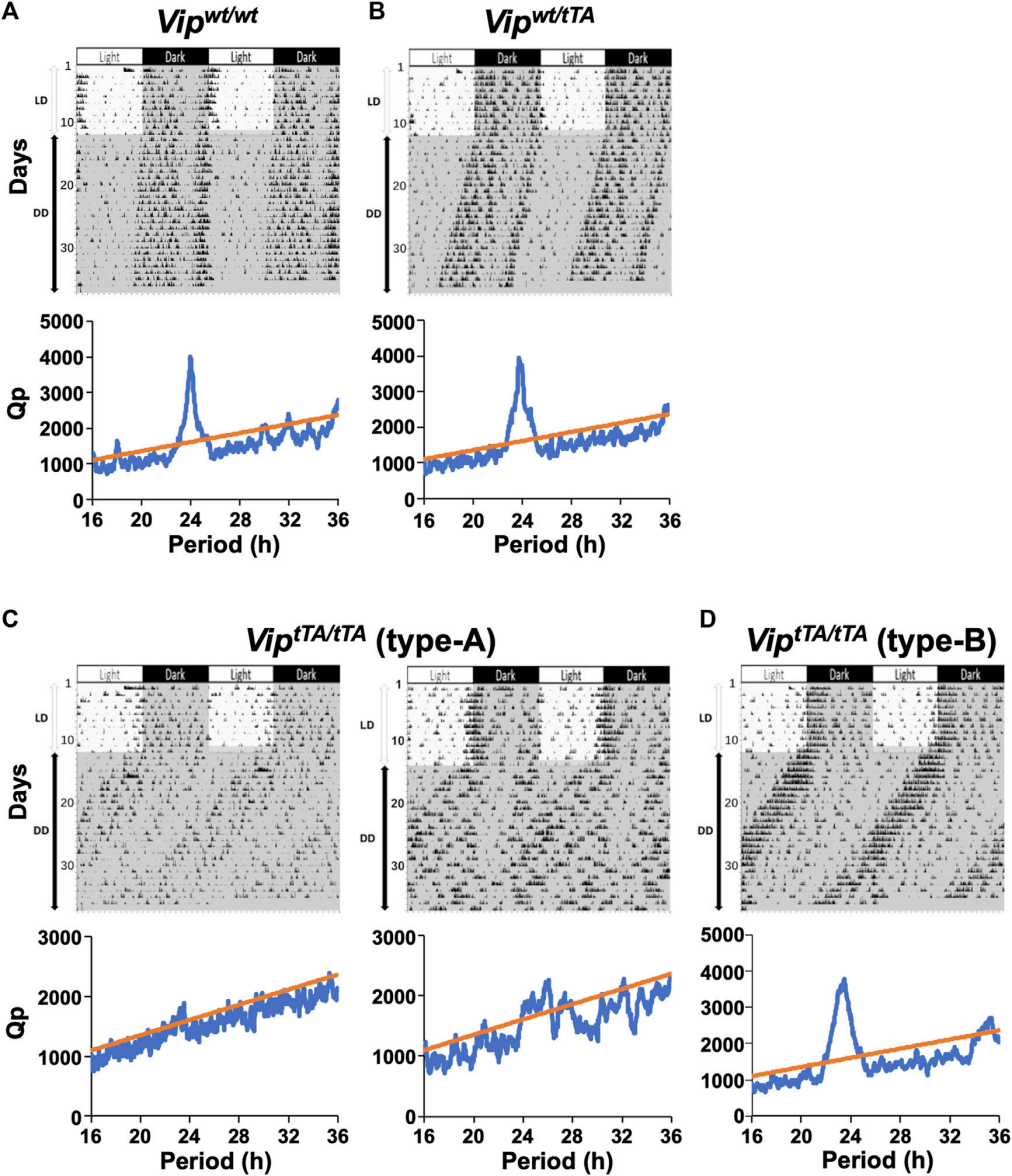
*Vip* ^*tTA/tTA*^ mice demonstrate a variety of abnormalities in circadian rhythmicity and synchrony. **(A–D)** Representative actograms and periodograms of the locomotor activity rhythm of one *Vip* ^*wt/wt*^ **(A)**, one *Vip* ^*wt/tTA*^ **(B)**, two type-A *Vip* ^*tTA/tTA*^ **(C)**, and one type-B *Vip* ^*tTA/tTA*^ mice. Animals were initially housed in 12:12 h light/dark (LD) conditions and then transferred to constant darkness (DD). Gray shadings in actograms indicate the time when lights were off. Periodograms are for the last 10 days in DD. Peaks above the diagonal line (indicating the 99.9% confidence level) between 16 and 36 h were considered significant circadian periods. *Vip* ^*wt/wt*^ and *Vip* ^*wt/tTA*^ mice show a clear dominant peak around 24 h **(A,B)**. Note that type-A *Vip* ^*tTA/tTA*^ mice expressed no clear period (i.e., arrhythmic; C left) or multiple periods (C right), whereas those of type-B showed more coherent circadian behavior, with a single, free-running period, although shortened **(D)**.

Previous studies of locomotor activity rhythm in *Vip* ^−/−^ mice have shown significantly attenuated circadian rhythmicity in constant darkness (DD), either with a shortened free-running period or multiple circadian periods (Colwell et al., 2003; Aton et al., 2005). A small number of *Vip* ^−/−^ mice even demonstrate arrhythmicity. Therefore, we next recorded the daily rhythm of spontaneous locomotor activity of *Vip* ^*wt/wt*^, *Vip* ^*wt/tTA*^, and *Vip* ^*tTA/tTA*^ mice. In these experiments, mice were individually housed in cages, first in a 12 h/12 h light/dark (LD) cycle for 10–15 days. Under this condition, all groups were entrained to the LD cycle and exhibited nocturnal rhythms in their activity (Figure 3).

**FIGURE 4:**
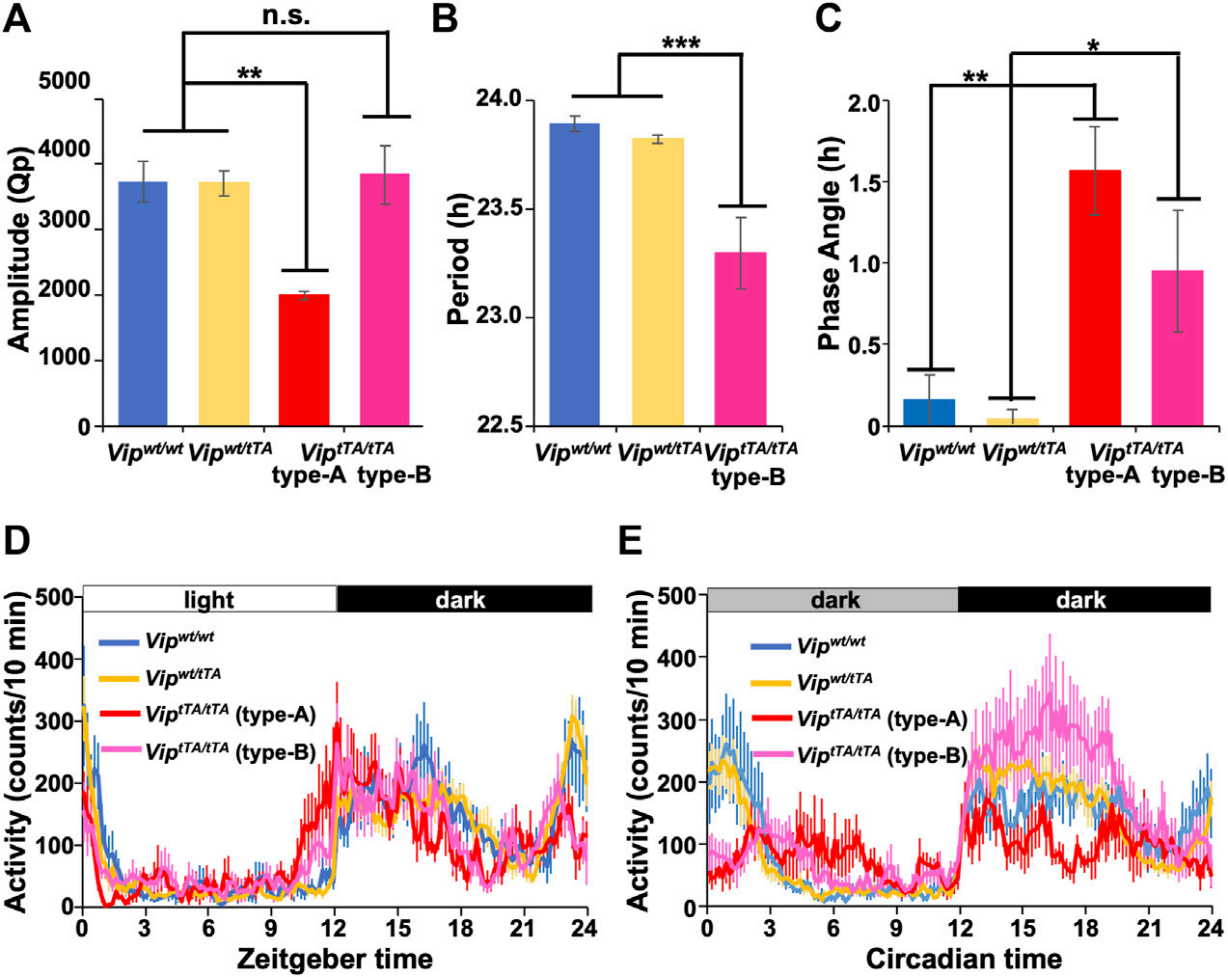
*Vip* ^*tTA/tTA*^ mice show impaired circadian rhythms. **(A)** The circadian amplitude of rhythms was significantly reduced in type-A *Vip* ^*tTA/tTA*^ mice (n = 5) compared to *Vip* ^*wt/wt*^ (n = 7), *Vip* ^*wt/tTA*^ (n = 12), and type-B *Vip* ^*tTA/tTA*^ (n = 7) mice. Bars show circadian amplitudes at the dominant period as determined by χ^2^ periodogram analysis (Qp values). **(B)** Free-running period was shortened in type-B *Vip* ^*tTA/tTA*^ mice. **(C)** Phase angle of entrainment under LD. **(D–E)** Daily profiles of circadian behavioral rhythms under LD **(D)** or DD **(E)**. Error bars indicate SEM. *, *p* < 0.05; **, *p* < 0.005; ***, *p* < 0.001 by one-way ANOVA followed by *post-hoc* Tukey tests.

Mice were then placed into DD, and their free-running rhythms were recorded. Under these conditions, several striking differences emerged in *Vip* ^*tTA/tTA*^ mice compared to *Vip* ^*wt/wt*^ and *Vip* ^*wt/tTA*^ mice. All *Vip* ^*wt/wt*^ and *Vip* ^*wt/tTA*^ mice free-ran with a single, stable circadian period (Figures 3A,B). The free-running periods were comparable between *Vip* ^*wt/wt*^ (23.89 ± 0.03 h) and *Vip* ^*wt/tTA*^ mice (23.82 ± 0.02 h) (Figure 4B). In contrast, among *Vip* ^*tTA/tTA*^ mice, one-half (5 of 12) exhibited multiple circadian periods or no statistically significant circadian period (type-A) (Figure 3C). The remaining half (7 of 12) free-ran with a single circadian period (type-B) (Figure 3D). However, the period of type-B *Vip* ^*tTA/tTA*^ mice (23.30 ± 0.16 h) was significantly shorter than that of *Vip* ^*wt/wt*^ mice (Figure 4B).

Type-A *Vip* ^*tTA/tTA*^ mice (Qp 1997.28 ± 61.92) also showed circadian amplitude significantly lower than that of *Vip* ^*wt/wt*^ mice (Qp 3,733.60 ± 306.41) in DD (Figure 4A). In contrast, there was no significant difference between type-B *Vip* ^*tTA/tTA*^ (Qp 3,840.01 ± 443.14) and *Vip* ^*wt/wt*^ mice. In addition, the circadian amplitude of *Vip* ^*wt/tTA*^ (Qp 3,706.82 ± 193.27) mice was not significantly different from *Vip* ^*wt/wt*^ mice (Figure 4A). The mean daily profiles of locomotor activity rhythm also demonstrated the attenuation of circadian rhythm of type-A *Vip* ^*tTA/tTA*^ mice both in LD and DD (Figures 4C,D). In contrast, type-B *Vip* ^*tTA/tTA*^ mice increased their locomotor activity during CT12∼18 (evening component) while they reduced that during CT21∼3 (morning component) in DD (Figure 4D). In LD, the activity onset was ∼1.5 h earlier in type-A and ∼0.8 h in type-B *Vip* ^*tTA/tTA*^ mice compared to other groups (Figures 3C,D and Figures 4C,D).

Based on the above data, we concluded that the circadian behavior rhythm of *Vip* ^*wt/tTA*^ mice was not significantly different compared to *Vip* ^*wt/wt*^ mice. However, *Vip* ^*tTA/tTA*^ mice were deficient in VIP expression and exhibited a variety of impairments in their circadian behavioral rhythms. The circadian phenotypes of *Vip* ^*tTA/tTA*^ mice were all consistent with the previous reports concerning *Vip* ^−/−^ mice (Colwell et al., 2003; Aton et al., 2005).

## Discussion

In this study, we generated *Vip* ^*tTA*^ knock-in mice in which tTA2-coding sequence was introduced into the endogenous *Vip* locus. As expected, the expression of tTA in *Vip* ^*wt/tTA*^ mice was highly specific to VIP neurons in the SCN. Therefore, in combination with transgenic mice or viral vectors with TRE-mediated transgene expression, *Vip* ^*tTA*^ mice can be used to express any protein specifically in VIP neurons. Furthermore, the double transgenic mice containing *Vip* ^*tTA*^ and *Avp-Cre* enabled us to simultaneously apply different genetic manipulations to VIP and AVP neurons in the SCN. Such a dual-targeting strategy would be potent for the study of SCN, a small but complicated neuronal network consisting of multiple types of neurons, including VIP neurons. A series of Cre driver mice specific to a particular type of SCN neurons are currently available, such as *Avp-Cre*, *Nms-Cre*, *Grp-Cre*, *Drd1a-Cre*, and *Vipr2-Cre* (Taniguchi et al., 2011; Lee et al., 2015; Mieda et al., 2015; Smyllie et al., 2016; Inoue et al., 2018; Cheng et al., 2019). Therefore, by crossing with one of them, *Vip* ^*tTA*^ mice would provide opportunities to directly examine the interactions between VIP neurons and another type of SCN neurons.

Homozygous *Vip* ^*tTA/tTA*^ mice were VIP-deficient and behaved similarly to *VIP* ^*−/-*^ mice (Colwell et al., 2003; Aton et al., 2005). The neuropeptide VIP, which is produced by a part of retinorecipient neurons of the SCN, has been demonstrated to be especially important for the maintenance and synchronization of cellular clocks in individual SCN neurons (Aton et al., 2005; Maywood et al., 2006). Therefore, attenuated oscillation and synchronization of SCN neurons may account for the arrhythmicity and multiple circadian periods observed in the half of *Vip* ^*tTA/tTA*^ mice (type-A). The short-period rhythmicity observed in type B *Vip* ^*tTA/tTA*^ mice is also a feature common to one-third of *VIP* ^*−/-*^ mice. However, the cause of their shortened period remains unclear. The advanced activity onset of *Vip* ^*tTA/tTA*^ mice in LD conditions may be due to their shortened circadian period and the reduction in photoentrainment, in which VIP signaling has been implicated (Colwell et al., 2003; Jones et al., 2015, 2018; Vosko et al., 2015). Thus, *Vip* ^*tTA/tTA*^ mice provide a novel model of VIP-deficiency in which we can target genetic manipulations to the putative VIP neurons. In contrast, heterozygous *Vip* ^*wt/tTA*^ mice were comparable to wild-type mice in the VIP expression and circadian behavior. Therefore, *Vip* ^*wt/tTA*^ mice enable us to target genetic manipulations to VIP neurons without influencing circadian behavior.

In summary, we generated a novel mouse line *Vip* ^*tTA*^. By using it, we can target exogenous gene expression to VIP neurons in both normal and VIP-deficient mice. In addition to the previously developed *Vip* ^*ires-Cre*^ and *Vip* ^*ires-Flp*^ mice (Taniguchi et al., 2011; He et al., 2016), *Vip* ^*tTA*^ mice are the third genetic tool based on the Tet system for manipulating VIP neurons. It will expand the opportunity for the genetic dissection of complex neuronal networks, such as the SCN, in combination with other genetic tools utilizing the Cre/loxP or FLP/FRT system.

## Data Availability

The original contributions presented in the study are included in the article, further inquiries can be directed to the corresponding author.
